# Automated Risser Grade Assessment of Pelvic Bones Using Deep Learning

**DOI:** 10.3390/bioengineering12060589

**Published:** 2025-05-29

**Authors:** Jeoung Kun Kim, Donghwi Park, Min Cheol Chang

**Affiliations:** 1 Department of Business Administration, School of Business, Yeungnam University, Gyeongsan-si 38541, Republic of Korea; kimjk70@yu.ac.kr; 2 Seoul Spine Rehabilitation Clinic, Ulsan-si 44607, Republic of Korea; bdome@hanmail.net; 3 Department of Rehabilitation Medicine, College of Medicine, Yeungnam University, Daegu 42415, Republic of Korea

**Keywords:** Risser grade, pelvic bone, bone age, radiograph, deep learning, artificial intelligence

## Abstract

(1) Background: This study aimed to develop a deep learning model using a convolutional neural network (CNN) to automate Risser grade assessment from pelvic radiographs. (2) Methods: We used 1619 pelvic radiographs from patients aged 12–18 years with scoliosis to train two CNN models—one for the right pelvis and one for the left. A multimodal approach incorporated 224 × 224-pixel regions of interest from radiographs, alongside patient age and gender. The models were optimized with Adam, weight decay, rectified linear unit (ReLU) activation, dropout, and batch normalization, while synthetic data augmentation addressed class imbalance. Performance was evaluated through accuracy, precision, recall, F1-score, and area under the receiver operating characteristic curve (ROC AUC). (3) Results: The right pelvis model achieved 83.64% accuracy; the left pelvis model reached 80.56%. Both models performed well for Risser Grades 0, 2, and 4, with the right pelvis model achieving a microaverage F1-score of 0.836 and ROC AUC of 0.895. The left pelvis model achieved a microaverage F1-score of 0.806 and ROC AUC of 0.872. Challenges arose from class imbalance in less frequent grades. (4) Conclusions: CNN models effectively automated Risser grade assessment, reducing clinician workload and variability.

## 1. Introduction

Bone age assessment (BAA) is a clinical approach that evaluates skeletal development stages in children [1,2,3]. BAA for boys and girls relies on findings from hand and elbow radiographs up to ages 15 and 13, respectively [1,2,3]. Beyond these ages, bone age is measured using ossification and fusion levels of iliac crest apophyses, following the Risser grading system, which includes the following stages [4,5]: Grade 0 indicates no ossification center at the level of an iliac crest apophysis; Grade 1 represents an apophysis under 25% of the iliac crest; Grade 2 corresponds to an apophysis over 25–50% of the iliac crest; Grade 3 indicates an apophysis over 50–75% of the iliac crest; Grade 4 reflects an apophysis over 75% of the iliac crest; and Grade 5 means complete ossification and fusion of the iliac crest apophysis. This classification provides a standardized method for assessing skeletal maturity in adolescents.

BAA with the Risser grading system presents several challenges that limit its efficiency and consistency in clinical practice [6]. The process is inherently time-consuming, necessitating detailed visual inspection and manual grading by trained radiologists or pediatricians. Additionally, BAA has low inter- (0.46) and intra-observer variability (0.49), meaning that different practitioners—or even the same practitioner at different times—may arrive at varying conclusions based on the same radiographic images [6]. Such subjectivity introduces uncertainty in clinical decisions, particularly when treatment plans hinge on precise skeletal maturity assessment.

Recent advancements in deep learning technology have revolutionized medical image analysis [7,8,9]. In particular, convolutional neural networks (CNNs) have shown significant promise in enabling automated diagnoses through complex pattern recognition in medical imaging [10,11,12]. Recent advancements in CNNs have significantly improved image tasks and focus on enhancing model performance by combining features from different layers or modalities, including transformer-based models, efficient architecture, multiscale feature fusion, and early and late fusion [10,11,12].

CNN models are extensively utilized in various medical applications. For instance, SpineNet employs intervertebral disk volumes as input and is trained with disk-specific class labels to automatically interpret the radiological grades from lumbar spine magnetic resonance images [13]. Furthermore, a multimodal CNN-based regression model has been developed to automatically perform BAA by learning from hand X-ray images in conjunction with patient age and gender. The developed model demonstrates robust overall performance in BAA. Specifically, it achieves an overall mean absolute error of 0.410 years, a root mean square error of 0.637 years, and an accuracy of 91.1% [3].

Recent CNN models, such as ConvNeXt, offer significant advantages for vision tasks by automatically learning hierarchical features and leveraging inherent spatial inductive biases. They contradict traditional ML models, such as support vector machine and random forest, which necessitate manual feature engineering and often lose spatial context. When compared to transformer models, the ConvNeXt CNN model retains crucial vision-specific inductive biases that can lead to good sample efficiency, while its architectural design promotes computational efficiency, providing a strong performance-to-resource ratio through optimized convolutional operations.

Feature fusion in a CNN enhances performance by aggregating information from diverse sources, such as multimodal data or different network layers [14,15,16]. This allows for a more comprehensive representation of input data, capturing both fine-grained details and the global context, leading to improved performance in various tasks [14,15,16]. We hypothesize that a CNN-based deep learning model can achieve comparable or superior accuracy in Risser grade assessment compared to traditional expert-based evaluation while significantly reducing assessment time and observer variability. Therefore, this study developed a deep learning algorithm using CNN to automatically determine Risser grades of pelvic bones and evaluated its efficacy.

## 2. Materials and Methods

### 2.1. Subjects

This study was approved by the Institutional Review Board of Yeungnam University Hospital (2024-06-005). The requirement for informed consent was waived by the institutional review board of Yeungnam University Hospital owing to the retrospective nature of the study. The data were accessed from 14 June 2024 to 31 December 2024. The authors had access to information that could identify individual participants during data collection.

The study cohort comprised patients aged 12–18 years who were confirmed to have scoliosis in the screening conducted at Yeungnam University Hospital from January 2010 to December 2022. Posteroanterior pelvic radiographs were utilized for algorithm development. The exclusion criteria were as follows: (I) radiographs with interference from extracorporeal objects and (II) radiographs with surgical implants. The radiographs were retrieved from the institution’s picture archiving and communication system, anonymized, and exported as JPEG images. A radiologist with over 20 years of clinical experience determined Risser grades from posteroanterior pelvic radiographs.

### 2.2. Deep Learning Model Development

This study developed two deep learning models for automated Risser grade assessment using Python 3.10.15 and TensorFlow 2.16.2 with Keras. To address the challenges posed by class imbalance and limited data, a multimodal approach incorporating both radiographic images and patient clinical data was implemented. Specifically, 224 × 224-pixel regions of interest from right and left pelvic radiographs, along with patient age and gender, were used as input. The synthetic minority oversampling technique (SMOTE) was used to address class imbalance [17], and traditional image augmentation techniques (tf.image random_flip_left_right, random_flip_up_down, and random_brightness with max_delta = 0.05) were applied to enhance data diversity.

Class imbalance, a prevalent challenge in medical research, can significantly hinder the performance of classification algorithms. SMOTE addresses this issue by augmenting the minority class through the generation of synthetic samples. Unlike simple duplication, which risks overfitting, SMOTE interpolates between existing minority instances and their k-nearest neighbors in the feature space. This process effectively expands the minority class representation, enhancing the classifier performance on imbalanced datasets and improving the prediction of rare yet clinically significant events.

**Figure 1:**
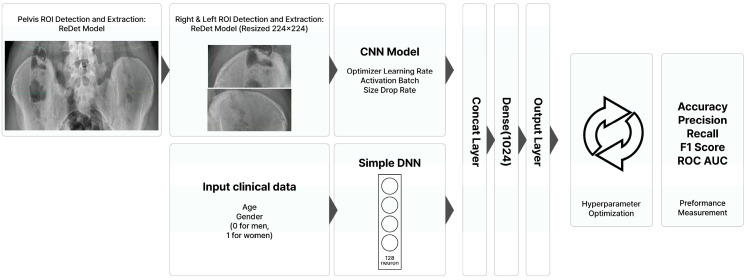
Summary of the model development process for assessing Risser grades.

The deep learning models determining Risser grades of the right and left pelvises utilized a ConvNextTiny CNN architecture [18], which was optimized with Adam with weight decay (AdamW) and rectified linear unit (ReLU) activation. Regularization was achieved through dropout and batch normalization. The system also employed a separate deep neural network (DNN) to process clinical data. Features extracted from the CNNs and DNN were fused using a concatenation layer, followed by additional dense layers culminating in an output layer generating Risser grade predictions. Furthermore, iterative hyperparameter optimization, guided by performance metrics (accuracy, precision, recall, F1-score, and area under the receiver operating characteristic curve [ROC AUC]), was conducted to refine the model performance. This integrated multimodal approach provides a comprehensive and automated pipeline for Risser grade assessment. Figure 1 provides an overview of the model development process for Risser grade determination.

### 2.3. Statistical Analyses

Statistical analyses were conducted using Python (version 3.10.15) with the Scikit-learn library (version 1.5.2) [19]. The diagnostic performance of the deep learning classification model for Risser grade assessment was evaluated by calculating accuracy, precision, recall, F1-score, and ROC AUC. These metrics were reported separately for models determining Risser grades on the right and left pelvises.

## 3. Results

A total of 1619 pelvic radiographs were used for the development of the deep learning model. The mean age of the subjects was 13.13 ± 1.73 years, comprising 582 males and 1037 females. Of these images, 1295 (80%) were used for training, whereas 324 (20%) were utilized for validation. The dataset exhibited a marked class imbalance, wherein the number of data for Risser Grade 4 was the largest (right and left sides: 42.1%), and that for Risser Grade 1 was the smallest (right side: 5.4%, left side: 5.8%). The specific ratios of each Risser grade are presented in the “Sample class size and ratio” columns of Table 1 and Table 2. During training, the model for determining Risser grades of the right pelvis (RT model) reached 100% accuracy while achieving a validation accuracy of 83.64%. In comparison, the model for the left side (LT model) obtained a training accuracy of 98.07% and a validation accuracy of 80.56%. Table 1 and Table 2 show the details of the developed models.

**Figure 2:**
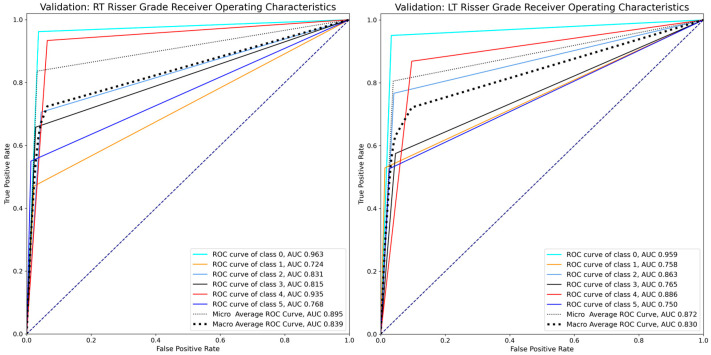
ROC AUC of the RT and LT models.

Evaluation metrics derived from the validation dataset indicated generally robust performances for both the RT and LT models. Specifically, the RT model demonstrated a microaverage F1-score of 0.836, a macroaverage F1-score of 0.727, and a microaverage ROC AUC of 0.895 (Figure 2). Meanwhile, the LT model achieved a microaverage F1-score of 0.806, a macroaverage F1-score of 0.708, and a microaverage ROC AUC of 0.872 (Figure 2).

**Figure 3:**
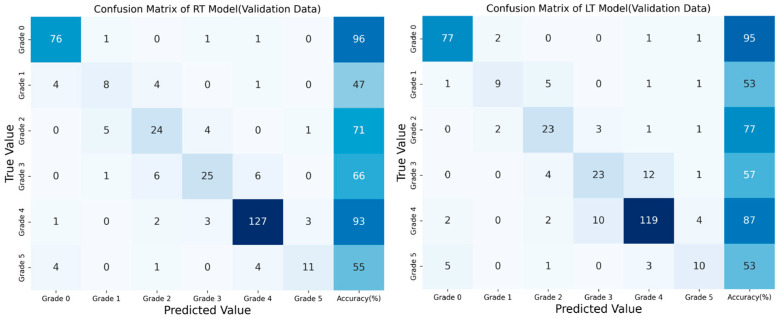
Confusion matrix of the RT and LT models.

The confusion matrices for the RT and LT models (Figure 3) revealed specific patterns in Risser grade classification. The RT model showed prediction accuracies of 96%, 71%, and 93% for Risser Grades 0, 2, and 4, respectively, with lower accuracies of 47%, 66%, and 55% for Grades 1, 3, and 5, respectively. Similarly, the LT model showed accuracies of 95%, 77%, and 87% for Grades 0, 2, and 4, respectively, with lower prediction accuracies of 53%, 57%, and 53% for Grades 1, 3, and 5. These matrices highlighted the impact of class imbalance. Both models demonstrated strong performance in identifying Risser Grades 0, 2, and 4. However, both models exhibited challenges in accurately classifying Grades 1, 3, and 5, which were often misclassified as adjacent grades. In particular, the models frequently assigned lower grades than the actual values for Grades 1 and 5, which had the least training data.

**Figure 4:**
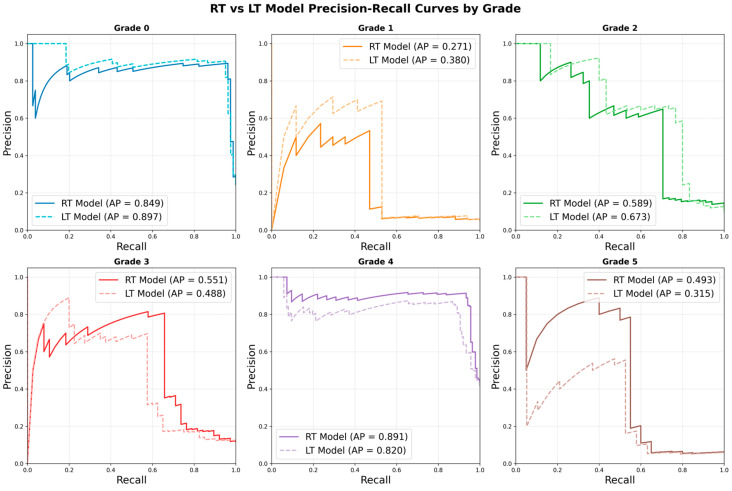
RT vs. LT model precision–recall curves by grade.

Furthermore, precision–recall analysis evaluated the RT and LT models across Grades 0–5, revealing significant grade-specific performance disparities (Figure 4). Both models achieved optimal performance in early skeletal maturity detection, with Grade 0 demonstrating the highest average precision scores (RT: 0.849, LT: 0.897) and Grade 1 maintaining relatively weak performance (RT: 0.271, LT: 0.380). However, intermediate grades (2–3) showed markedly reduced performance, with Grade 4 exhibiting particularly superior results (RT: 0.891, LT: 0.820), while Grade 5 demonstrated relatively weak performance (RT: 0.493, LT: 0.315).

## 4. Discussion

Herein, we developed an automated deep-learning-based system for assessing Risser grades of pelvic bones using CNN to analyze radiographs and patient clinical data. The results demonstrated the potential of deep learning in assessing skeletal maturity using the Risser grading system, traditionally reliant on expert assessment. The model showed good performance in determining Risser grades, especially for the more common grades (Risser Grades 0, 2, and 4).

Regarding the performance of our deep learning model for both right and left pelvic radiographs, the RT model achieved a validation accuracy of 83.64%, whereas the LT model had a validation accuracy of 80.56%, indicating that the models can reliably predict Risser grades. Evaluation metrics such as F1-score, precision, recall, and ROC AUC revealed the models’ overall good classification performance, with the RT and LT models achieving microaverage F1-scores of 0.836 and 0.806 and microaverage ROC AUC of 0.895 and 0.872, respectively, indicating good model sensitivity and specificity [20,21].

This study identified class imbalance in the dataset as a major challenge. Risser Grade 4 was the most prevalent class, representing a substantial portion of the training data (42.1% for both right and left pelvic images). Conversely, the data portion for Risser Grade 1 was low at 5.4% for the right pelvic images and 5.8% for the left pelvic images. This imbalance caused misclassification, particularly for Risser Grades 1, 3, and 5, which were frequently predicted as adjacent grades. The lower accuracies for these grades (i.e., 47%, 66%, and 55% for Risser Grades 1, 3, and 5, respectively, in the RT model and 53%, 57%, and 53% for Risser Grades 1, 3, and 5, respectively, in the LT model) seemed to be due to their lower amount of data compared to Risser Grades 0, 2, and 4. Despite the use of SMOTE to address the data imbalance by generating synthetic samples for underrepresented classes, the models still tended to classify less common grades as more prevalent ones. These findings highlight the need for a balanced dataset to enhance the model’s accuracy in classifying less frequent Risser grades.

Several studies have addressed class imbalance using focal loss, cost-sensitive learning, or ensemble methods [22,23,24]. Additionally, Kim et al. developed a deep learning model that predicts bone age in hand radiographs [3]. They showed that a multimodal regression-based model integrating radiographic and clinical data can achieve high accuracy (91.1%), despite significant imbalance in gender distribution (2162 females vs. 812 males). We addressed the data imbalance issue using SMOTE, which generates synthetic examples for minority classes by interpolating between existing minority class samples. This approach helps balance class distribution without simply duplicating data, thereby reducing the risk of overfitting [19]. Furthermore, SMOTE is known to enhance classifier performance on underrepresented classes by providing a more diverse and informative training set [19].

The development of an automated system for Risser grade assessment has important clinical implications. As the manual measurement of Risser grading is time-consuming, highly dependent on physician experience, and prone to interobserver variability [25], automating this process using deep learning models can help reduce the workload of clinicians and provide more consistent and accurate assessments.

A limitation of this study is the residual overfitting in our models, despite employing mitigation strategies such as early stopping, regularization (dropout and batch normalization), and SMOTE-based data augmentation. The persistent discrepancy between training and validation accuracies for both the RT and LT models indicates limited generalization to unseen data. This factor warrants consideration when interpreting the study’s findings.

To enhance the model’s classification of Risser grades accurately, it is necessary to balance datasets by increasing representation of less frequent grades or employing advanced oversampling techniques. Although our model currently determines Risser grades, future research should focus on developing a model to assess potential height increase using longitudinal data on height change. In addition, the dataset used for model training is limited to a single institution, potentially reducing the model’s generalizability to other patient populations or institutions. Additionally, a larger amount of training data can improve the model’s performance. Moreover, a study comparing time saved using the developed model versus manual assessment would demonstrate the model’s effectiveness.

## 5. Conclusions

Our study demonstrated the potential of deep learning models, particularly CNN-based architectures, for automating the assessment of Risser grades in pelvic radiographs. The right pelvis model achieved an accuracy of 83.64%, with a microaverage F1-score of 0.836 and ROC AUC of 0.895. The left pelvis model reached an accuracy of 80.56%, with a microaverage F1-score of 0.806 and ROC AUC of 0.872. Both models performed well for Risser Grades 0, 2, and 4. The model performed well in identifying common Risser grades, but addressing class imbalance is necessary to improve accuracy across all grades. Overall, the findings suggest that deep learning can effectively enhance the efficiency and consistency of BAA in clinical practice.

## Figures and Tables

**Table 1:** Details of the model for determining Risser grades of the right pelvis (RT model).

Sample size and ratio Sample class size and ratio	- 80% for training: 1295; 20% for validation: 324; total: 1619 - Class 0, 394 (24.3%); Class 1, 94 (5.8%); Class 2, 158 (9.8%); Class 3, 194 (12%); Class 4, 682 (42.1%); Class 5, 97 (6%)					
Gender ratio Age distribution	- Male: 582 (35.9%); Female: 1037 (64.1%) - 9 (42, 2.59%); 10 (80, 4.94%); 11 (161, 9.94%); 12 (285, 17.6%); 13 (349, 21.56%); 14 (308, 19.02%); 15 (236, 14.58%); 16 (133, 8.21%); 17 (23, 1.42%); 18 (2, 0.12%)					
RT model	- ConvNextTiny CNN model - AdamW optimizer, ReLU activation, batch size 16 - Image input: right pelvis ROI (224 × 224) - Clinical data for input: age and gender - Dropout and batch normalization layer for regularization - Training accuracy: 100%; validation accuracy: 83.64%					
Model performance	Class	Precision	Recall	F1-score	Support	ROC AUC
(Validation data)	0	0.894	0.962	0.927	79	0.963
	1	0.533	0.472	0.500	17	0.724
	2	0.649	0.706	0.676	34	0.831
	3	0.758	0.658	0.704	38	0.815
	4	0.914	0.934	0.924	136	0.935
	5	0.733	0.550	0.629	20	0.768
	Microaverage	0.836	0.836	0.836	324	0.895
	Macroaverage	0.747	0.713	0.727	324	0.839

CNN, convolutional neural network; AdamW, Adam with weight decay; ReLU, rectified linear unit; ROI, region of interest; ROC, receiver operating characteristic; AUC, area under the curve.

**Table 2:** Details of the model for determining Risser grades of the left pelvis (LT model).

Sample size and ratio Sample class size and ratio	- 80% for training: 1295; 20% for validation: 324; total: 1619 - Class 0, 402 (24.8%); Class 1, 87 (5.4%); Class 2, 149 (9.2%), Class 3, 202 (12.5%); Class 4, 682 (42.1%); Class 5, 97 (6%)					
Gender ratio Age distribution	- Male: 582 (35.9%); Female: 1037 (64.1%) - 9 (42, 2.59%); 10 (80, 4.94%); 11 (161, 9.94%); 12 (285, 17.6%); 13 (349, 21.56%); 14 (308, 19.02%); 15 (236, 14.58%); 16 (133, 8.21%); 17 (23, 1.42%); 18 (2, 0.12%)					
LT model	- ConvNextTiny CNN model - AdamW optimizer, ReLU activation, batch size 32 - Image input: left pelvis ROI (224 × 224) - Clinical data for input: age and gender - Dropout and batch normalization layer for regularization - Training accuracy: 98.07%; validation accuracy: 80.56%					
Model performance	Class	Precision	Recall	F1-score	Support	ROC AUC
(Validation data)	0	0.906	0.951	0.928	81	0.959
	1	0.692	0.529	0.600	17	0.758
	2	0.657	0.767	0.708	30	0.863
	3	0.639	0.575	0.605	40	0.765
	4	0.869	0.869	0.869	137	0.886
	5	0.556	0.526	0.541	19	0.750
	Microaverage	0.806	0.806	0.806	324	0.872
	Macroaverage	0.720	0.703	0.708	324	0.830

CNN, convolutional neural network; AdamW, Adam with weight decay; ReLU, rectified linear unit; ROI, region of interest; ROC, receiver operating characteristic; AUC, area under the curve.

## Data Availability

The datasets generated during and/or analyzed during the current study are available from the corresponding author on reasonable request.

## Abbreviations

CNN: conventional neural network; ROI: region of interest; SMOTE: synthetic minority oversampling technique; AdamW: Adam with weight decay; ReLU: rectified linear unit; DNN: deep neural network; ROC AUC: area under the receiver operating characteristic curve.
